# Lactate as a biomarker for discriminating and assessing prognosis of neonatal necrotising enterocolitis: A systematic review and meta-analysis

**DOI:** 10.12669/pjms.42.6.16103

**Published:** 2026-06

**Authors:** Haiping Shen, Yuhong Lu

**Affiliations:** 1 Haiping Shen, Department of Neonatology, Huzhou Maternity & Child Health Care Hospital, 2 East Street, Huzhou, Zhejiang Province 313000, P.R. China; 2 Yuhong Lu, Department of Neonatology, Huzhou Maternity & Child Health Care Hospital, 2 East Street, Huzhou, Zhejiang Province 313000, P.R. China

**Keywords:** Biomarker, Diagnosis, Lactic acid, Mortality, Neonatal necrotising enterocolitis

## Abstract

**Background and Objective:**

Neonatal necrotising enterocolitis (NEC) is a severe neonatal gastrointestinal disease associated with high morbidity and mortality. The present systematic review and meta-analysis examined if blood lactate could be used in the discrimination and prognosis of NEC.

**Methodology:**

PubMed, Embase, Scopus, and Web of Science databases were searched for relevant studies from the database inception to 26 January 2026. Diagnostic studies comparing lactate levels between NEC and control groups were pooled using standardised mean differences (SMDs). Prognostic studies evaluating mortality were pooled using odds ratios (ORs) under a random-effects model.

**Results:**

Nine studies were included, comprising four diagnostic and five prognostic studies. Lactate levels were significantly higher in neonates with NEC compared with controls (SMD = 1.66, 95% CI 0.78–2.55 I^2^ = 93%). In prognostic analyses, higher lactate levels were associated with an increased risk of mortality, but the pooled effect did not reach statistical significance (OR = 1.30, 95% CI 0.92–1.83; I^2^ = 75.8%). In the sensitivity analysis, excluding one study led to a statistically significant association between higher lactate levels and NEC mortality.

**Conclusions:**

This review suggests that lactate levels are significantly higher in neonates with NEC as compared with controls. However, while elevated lactate tends to be associated with increased mortality risk, substantial heterogeneity and limited evidence preclude its use as a standalone prognostic marker. Further research is needed to strengthen the present conclusions.

***Registration No:*** PROSPERO (CRD420261292430).

## INTRODUCTION

Necrotising enterocolitis (NEC) is a critical and severe gastrointestinal emergency mostly seen in preterm and low-birth-weight neonates.^1^ Despite important advancements in neonatal intensive care, NEC has been a major cause of mortality and morbidity. Mortality rates in severe NEC cases can approach 20–30%.^2^ The clinical course of NEC is often unpredictable, and is often accompanied by nonspecific initial symptoms which can rapidly progress to intestinal necrosis, sepsis, and multiorgan failure.^3^,^4^ The heterogeneity in presentation requires researchers to develop reliable biomarkers for early diagnosis and effective risk stratification.^5^ The pathophysiology of NEC is multifactorial and complex. A number of factors are involved like intestinal immaturity, dysregulated inflammatory response, microbial imbalance, and impaired tissue perfusion.^6^ Research has noted that intestinal ischemia and systemic hypoperfusion are important contributors to the pathogenesis and progression of the disease.^7^ Impaired oxygen delivery causes anaerobic metabolism and lactate accumulation, which can be an indicator of cellular hypoxia and metabolic stress.^8^ In this context, blood lactate levels have gained importance in recent time, as a readily available biomarker that may be used to assess the severity of NEC.

Recent studies have shown that neonates with NEC have elevated lactate levels compared with non-NEC controls, indicating that lactate may serve as an adjunctive biomarker to identify affected infants.^9^ Nonetheless, differences in the included patient cohorts, timing of lactate assessment, and analytical techniques has led to variable results, thereby preventing stong evidence on the discriminative ability of lactate in NEC. In addition to this, lactate has also been explored as a prognostic indicator. Multiple cohort studies have indicated that higher lactate concentrations are correlated with increased mortality, with non-survivors showing high levels.^10^,^11^

However, the robustness and independence of this association has remained uncertain in literature. To date, no detailed quantitative synthesis is available that has evaluated the discriminative and prognostic relevance of lactate in neonatal NEC. Since lactate is an easily available investigation, it can be used by both clinicians and nursing personnel for rapid discrimination and prognosis of NEC. Therefore, this systematic review and meta-analysis aimed to compare lactate levels in neonates diagnosed with NEC to those in control populations and to examine the association between elevated lactate levels and mortality.

## METHODOLOGY

Preferred Reporting Items for Systematic Reviews and Meta-Analyses (PRISMA) guidelines were followed in the reporting of this review.^12^ The review was registered on PROSPERO (CRD420261292430).

### Eligibility criteria:

We included observational analytical studies assessing the discriminative ability and prognostic significance of lactate levels in NEC. Included studies were those which included neonates diagnosed with NEC based on standardized clinical or radiological criteria and reported blood lactate concentrations. For assessing discriminative ability, studies were to compare lactate levels between NEC-affected neonates and NEC non-affected control groups. In case of prognostic evaluation, studies were to report mortality data based on lactate levels, either by comparing survivors and non-survivors or by providing effect size by means of odds ratios (ORs) or hazard ratios (HRs). Both prospective and retrospective cohort and case–control designs were eligible. We excluded review articles, conference abstracts, studies without sufficient quantitative data, animal studies, and studies with overlapping populations.

### Search and selection of studies:

A comprehensive systematic search was performed by two reviewers independently across the four major electronic databases of PubMed, Embase, Scopus, and Web of Science, from their inception to 26 January 2026. Details of the search strategy are shown in Supplementary Table-I. The reference lists of included articles were also manually screened to identify further important studies. Following the removal of duplicates, titles and abstracts of remaining studies were independently reviewed by two investigators (HS & YL). The full-text evaluation was then conducted for studies found fit to be eligible. Discrepancies regarding study inclusion were resolved via discussion and consensus. Studies fulfilling the predefined eligibility criteria were then classified into those assessing discriminative ability and prognosis.

**Table-SI:** Search Strategy for Databases.

**PubMed** ((“Enterocolitis, Necrotizing”[Mesh] OR “necrotizing enterocolitis” OR NEC) AND (neonate OR newborn OR infant)) AND (lactate OR lactic acid OR hyperlactatemia)
**Embase** (‘necrotizing enterocolitis’/exp OR ‘necrotizing enterocolitis’ OR NEC) AND (neonate OR newborn OR infant) AND (‘lactic acid’/exp OR lactate OR hyperlactatemia)
**Scopus** (TITLE-ABS-KEY(“necrotizing enterocolitis” OR NEC) AND TITLE-ABS-KEY(neonate OR newborn OR infant) AND TITLE-ABS-KEY(lactate OR “lactic acid” OR hyperlactatemia))
**Web of Science** (TS=(“necrotizing enterocolitis” OR NEC) AND TS=(neonate OR newborn OR infant) AND TS=(lactate OR “lactic acid” OR hyperlactatemia))

### Data management:

Data were independently collected by two reviewers utilizing a standardized data extraction form. Variables extracted encompassed study characteristics, including author, publication year, country, and study design, population details, NEC definition, sample size, gestational age, birth weight, timing and modality of lactate measurement, lactate concentrations and their units, as well as reported effect estimates concerning mortality. In studies on discriminative ability, lactate data were extracted for both the NEC and control groups. In studies on prognosis, data were obtained separately for survivors and non-survivors. When lactate values were reported as medians with interquartile ranges or ranges, they were converted to means and standard deviations using established statistical methods.^13^ Lactate units were standardized to mmol/L where necessary.

### Risk of bias analysis:

The methodological quality and risk of bias of the included studies were independently assessed by two reviewers using the Newcastle-Ottawa Scale.^14^ Studies were assessed for selection of cohort, comparability and outcome assessment. Two reviewers conducted the process and resolved disagreements through discussion.

### Statistical analysis:

Meta-analyses were conducted in ‘R’ software. We used the random-effects model using the DerSimonian–Laird method. In studies examining discriminative ability, standardized mean differences (SMDs) with Hedges’ correction and 95% confidence intervals (CIs) were calculated to compare lactate levels between NEC and control groups. For studies on prognosis, ORs with 95% CIs were used to pool data. When HRs were reported, they were assumed to be equivalent to ORs to facilitate exploratory data pooling. Effect estimates were log-transformed prior to analysis. Statistical heterogeneity was examined by the Cochran’s Q test and I^2^ statistic. Sensitivity analyses was done by removing each study one at a time to assess the stability of the pooled estimates. Publication bias was not assessed due to limited number of studies in each analysis. Certainty of evidence was assessed by GRADE approach.

## RESULTS

### Search results:

**Fig.1:**
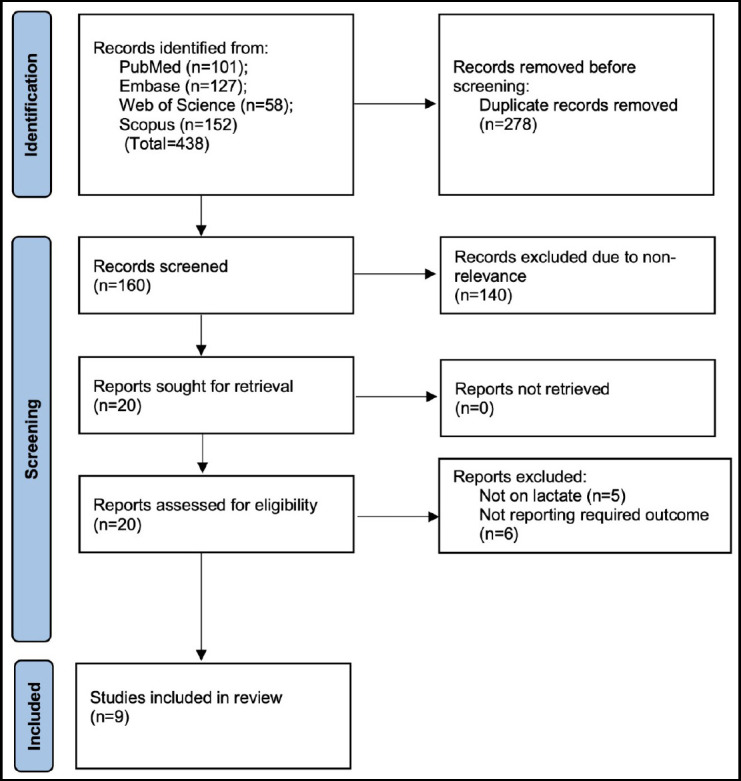
Study flowchart.

There were 438 studies found from the four databases. After removing duplicates, 160 studies remained. Twenty studies were selected for full text analysis of which nine met the inclusion criteria^9^-^11^,^15^-^20^ (Fig.1).

### Study characteristics:

The baseline characteristics of the studies are shown in Table-I and Table-II. Most studies used a retrospective cohort design. NEC was mostly defined based on modified Bell’s staging criteria. The control group in the studies consisted of neonates without NEC. In studies on discriminative ability, lactate levels were measured at different time points. Prognostic studies evaluated lactate either as a continuous variable or using study-specific thresholds. Based on the Newcastle–Ottawa Scale, the included studies had low to moderate risk of bias, with scores ranging from six to nine (Supplementary Table-II).

**Table-I:** Baseline data of studies assessing discriminative value of lactate in NEC.

Baseline variable	Karakurt 2026		Li 2025		Ahmed 2020		Lei 2016	
Country	Turkey		China		Egypt		China	
Study design	Retrospective cohort		Retrospective cohort		Prospective cohort		Prospective cohort	
Group	NEC	Control	NEC	Control	NEC	Control	NEC	Control
Sample size (n)	27	90	71	167	55	23	27	69
Gestational age (weeks)	28.1 ± 2.1	28.4 ± 2.3	31.9 (29.6–33.7)	33.1 (30.7–35.4)	33.38 ± 3.39	35.30 ± 3.86	35.1 ± 2.6	34.3 ± 1.7
Birth weight	1020 ± 240 g	1045 ± 260 g	1405 (1110–1840) g	1800 (1355–2270) g	NR	NR	1898.4 ± 285.3 g	1845.7 ± 267.5 g
Male sex (%)	55.6	53.3	66.2	59.9	63.6	65.2	59.3	56.5
Postnatal age at sampling	NR	NR	Day 1	Day 1	16.85 ± 6.34 days	14.30 ± 6.41 days	15.1 ± 1.8 days	12.3 ± 2.9 days
NEC definition	Bell stage ≥ II	NA	Bell stage ≥ II	NA	Bell stages I–III	NA	Bell stage II–III	NA
Sample type	Arterial blood gas	Arterial blood gas	Blood	Blood	Venous blood	Venous blood	Venous blood	Venous blood
Timing of lactate measurement	Postnatal days 1–3	Postnatal days 1–3	Day 1 of life	Day 1 of life	At diagnosis	At enrollment	Within 24 h of NEC onset	Weeks 2–3 of life
Lactate value* mmol/L	5.2 ± 4.7	4 ± 3	3.1 ± 1.26	1.5 ± 0.74	3.59 ± 0.84	1.70 ± 0.24	0.39 ± 0.32	0.04 ± 0.02

NEC, necrotizing enterocolitis; NR, not reported; NA, not applicable.

* Median (Interquartile) data was converted into mean and standard deviation.

**Table-II:** Baseline data of studies assessing the prognostic value of lactate in NEC.

Baseline variable	Han 2025		Huang 2025		Kuik 2022		Wang 2023		Kordasz 2022	
Country	China		China		Netherlands		China		Switzerland	
Study design	Retrospective cohort		Retrospective cohort		Retrospective cohort		Retrospective cohort		Retrospective cohort	
Group	Survivors	Non-survivors	Survivors	Non-survivors	Survivors	Non-survivors	Survivors	Non-survivors	Survivors	Non-survivors
NEC definition	Bell II–III	Bell II–III	Bell ≥ IIb	Bell ≥ IIb	Bell ≥ II	Bell ≥ II	ICD-10 P77.x	ICD-10 P77.x	Bell ≥ II	Bell ≥ II
Sample size	129	39	41	18	11	11	95	9	129	28
Gestational age (weeks)	32 (26–40)	30 (25–39)	NR	NR	26.9 (26.3–29.3)	27.0 (26.1–28.3)	NR	NR	32.0 (5.1)	27.0 (5.3)
Birth weight	1630 (800–3950) g	1340 (610–2800) g	NR	NR	900 (750–1250) g	1105 (670–1170) g	2.08 (1.69–2.75) kg	1.70 (1.37–1.98) kg	1480 (750) g	855 (955) g
Male sex (%)	62.8	64.1	NR	NR	55	55	65	89	NR	NR
Age at diagnosis / onset	12 (1–72) d	14 (1–83) d	NR	NR	9 (6–12) d	9 (6–12) d	NR	NR	8 (11) d	11 (13.2) d
Timing of lactate measurement	≤24 h pre-surgery	≤24 h pre-surgery	At NEC diagnosis	At NEC diagnosis	≤24 h pre-surgery	≤24 h pre-surgery	Worst ICU value	Worst ICU value	At onset / during NEC	At onset / during NEC
Lactate value (mmol/L)	2.2 (0.7–11.2)	4.1 (1.6–15.0)	5.8 (4.1–6.9)	7.9 (6.3–9.8)	1.1 (1.0–1.6)	4.6 (2.8–8.0)	2.75 (2.10–3.58)	5.10 (3.00–19.00)	2.1 (1.8)	3.95 (4.38)
Lactate cutoff / modeling	3.15 mmol/L	3.15 mmol/L	5.4 mmol/L	5.4 mmol/L	Continuous	Continuous	Per 1 mmol/L	Per 1 mmol/L	3.8 mmol/L	3.8 mmol/L
Effect estimate	Reference	HR 1.18 (1.05–1.32)	Reference	OR 1.70 (1.08–2.66)	Reference	OR 0.55 (0.32–0.97)	Reference	OR 1.45 (1.05–2.00)	Reference	OR 3.8 (1.4–10.0)
Adjusted analysis	Yes	Yes	Yes	Yes	Yes	Yes	Yes	Yes	No	No

NR, not reported; d, days; ICD, international classification of diseases.

**Table-SII:** Risk of bias assessment of included studies using the Newcastle–Ottawa Scale (NOS).

Study	Selection (★/4)	Comparability (★/2)	Outcome / Exposure (★/3)	Total score (★/9)
Lei 2016	★	★	★	6
Ahmed 2020	★	★	★	6
Li 2025	★	★	★	8
Karakurt 2026	★	★	★	7
Han 2025	★	★	★	9
Huang 2025	★	★	★	7
Kuik 2022	★	★	★	6
Wang 2023	★	★	★	7
Kordasz 2022	★	★	★	7

### Diagnostic value of lactate in NEC:

**Fig.2:**
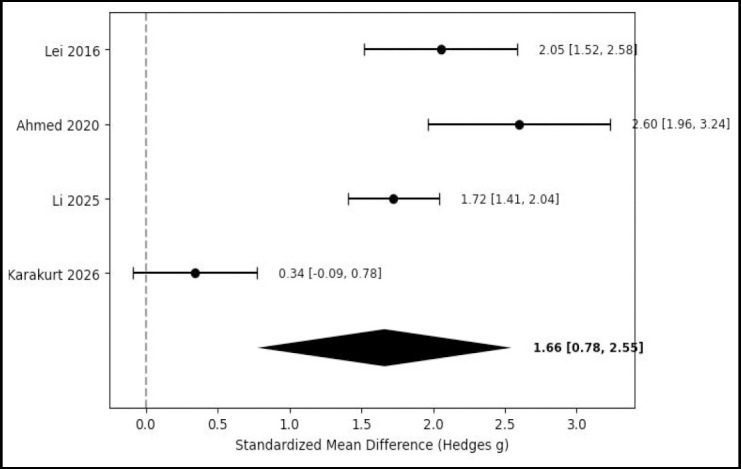
Meta-analysis of lactate levels between NEC and controls.

Four studies evaluated lactate levels in neonates with NEC compared with control populations. In the random-effects meta-analysis, lactate levels were significantly higher in neonates with NEC compared with controls (SMD = 1.66, 95% CI 0.78–2.55). However, heterogeneity was substantial (I^2^ = 93%) (Fig.2). On sensitivity analysis, no change in the significance of results was seen. Certainty of evidence was very low (Supplementary Table-III).

**Table-SIII:** GRADE assessment of evidence.

	Lactate levels	Prognostic ability
No. of studies	4	5
***Downgrade quality of evidence***		
Risk of bias	Very serious*	Very serious*
Inconsistency	No	No
Indirectness	No	No
Imprecision	Serious^	No
***Publication bias***		
***Upgrade quality of evidence***		
Large effect	No	No
Plausible confounding	No	No
Dose-response	No	No
Overall certainty of Evidence	Very low	Very low

* NOS score ranged from 6-8 for majority included studies, ^Wide Confidence intervals.

### Prognostic value of lactate for mortality in NEC:

**Fig.3:**
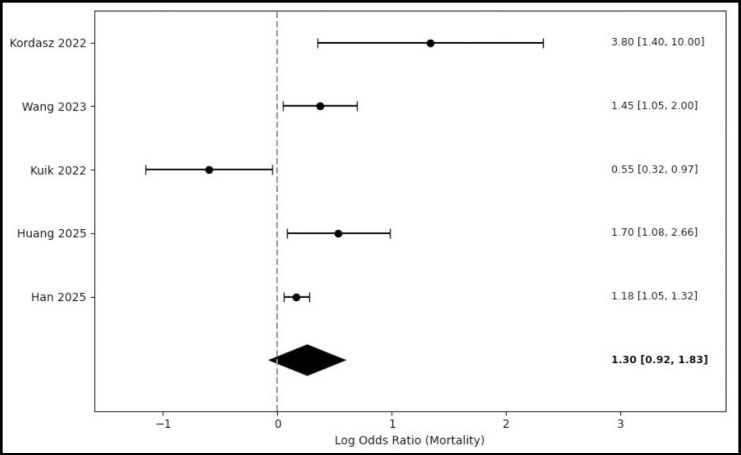
Meta-analysis of prognostic ability of lactate in predicting mortality in NEC.

Five studies examined the association between lactate levels and mortality in neonates with NEC. In the random-effects meta-analysis, higher lactate levels were associated with an increased risk of mortality. However, the pooled effect did not reach statistical significance (OR = 1.30, 95% CI 0.92–1.83). Substantial heterogeneity was observed (I^2^ = 75.8%) (Fig.3). During sensitivity analysis, exclusion of the study of Kuik et al.^18^ resulted in a statistically significant association between high lactate levels and mortality in NEC (OR = 1.48, 95% CI 1.10–1.99, I^2^ = 64%). Certainty of evidence was very low (Supplementary Table-III).

## DISCUSSION

This review is the first in literature to examine the discriminative and prognostic importance of lactate levels in NEC. Our results show that neonates with NEC have a significantly higher lactate level as compared to controls. This supports the hypothesis that lactate could be a biomarker of intestinal hypoperfusion and metabolic stress seen with NEC. Nonetheless, the findings should be interpreted with caution due to the high heterogeneity in the included studies. Although elevated lactate levels were generally found to be associated with higher risk of mortality in NEC, the combined effect size did not achieve statistical significance. Notably, sensitivity analysis showed that after excluding one study^18^, there was a statistically significant association between elevated lactate levels and mortality.

NEC often has an insidious onset, lacking specific diagnostic methods. The disease progresses rapidly, causing serious complications in a short period.^7^ Consequently, identifying risk factors during the prenatal and early neonatal stages is important for developing strategies to reduce morbidity and mortality.^4^,^5^ In this context, blood-based biomarkers could be important for both detecting and prognosticating NEC.^10^ Early signs of NEC often similar to those of sepsis or feeding intolerance, which limits the accuracy of clinical examination and radiographic findings in the initial phases for accurate diagnosis.^5^ Circulating biomarkers can provide an objective and easily accessible method to detect the underlying physiological processes of NEC like reduced blood flow, inflammation, and metabolic stress, often before clinical symptoms become apparent. These markers may also provide information on the disease severity, response to treatment, and the risk of adverse outcomes, including the need for surgery or mortality.^18^,^19^ The advantage of blood-based biomarkers is that they can be measured repeatedly at the bedside, allowing clinicians to monitor disease progression and promptly adjust treatment strategies. Of the various markers, lactate is particularly valuable due to its widespread availability and established role in critical illness.^21^,^22^

The current study has provided high-quality evidence on the importance of lactate in NEC. It was noted that lactate could be a potential biomarker for both discriminating and assessing prognosis NEC. The sensitivity analysis also indicated that higher lactate levels may be associated with an increased risk of mortality in NEC. The role of lactate noted in NEC is similar to other critical conditions, like sepsis, mesenteric ischaemia, and shock states, wherein lactate is a marker of tissue hypoxia and disease severity rather than identifying the disease per se.^21^,^23^ Lactate is among the most frequently used biomarkers for sepsis diagnosis, especialy in the emergency departments. Guidelines have suggested that lactate levels may be a proxy for assessing the severity of circulatory dysfunction and monitoring the progress of resuscitation.^21^ However, as a biomarker, lactate lacks specificity because numerous factors can affect its systemic levels, and therefore guidelines only provide weak recommendations for its use.^24^ Literature also shows that the median serum lactate level is higher in patients with acute mesenteric ischaemia than in those with non-ischaemic disease, and lactate levels may be used to assess the prognosis in these patients.^23^,^25^ A recent study by Lau et al.^22^ has shown that lactate can help predict mortality in patients with necrotising fasciitis, and combining it with albumin may provide even better predictive performance.

A key finding of this meta-analysis was the high heterogeneity across studies in both the analyses. This heterogeneity is mostly from the significant clinical and methodological differences seen in the included studies, like variations in study populations (preterm-only versus cohorts with mixed gestational ages), disease severity, case definitions, timing of lactate assessment, and the the methods used to model lactate levels (continuous variables or threshold-based cut-offs). Such heterogeneity is expected, in view of the typically abrupt disease onset and highly variable clinical course. The substantial heterogeneity suggests that the pooled estimates should be regarded as indicative of the overall direction and magnitude of effects and should be interpreted with caution. Although conducting subgroup and meta-regression analyses could have provided information on the potential sources of heterogeneity, the limited number of eligible studies prevented such stratified analyses. Therefore, our inability to perform more detailed analyses reflects an inherent limitation of the available evidence rather than a methodological flaw of this review.

The high lactate levels in NEC is reflective of the various pathophysiological mechanisms associated with the onset and progression of NEC. Intestinal ischemia combined with compromised systemic perfusion are hallmarks of NEC. They cause a metabolic shift toward anaerobic pathways, leading to increased lactate production at the cellular level.^11^ Disruption of the immature microcirculation within the intestine further worsens the mucosal hypoxia, causing epithelial damage, disturbed barrier function, and bacterial translocation, thereby elevating the inflammatory and metabolic stress response.^25^ The rising lactate concentrations may also serve as early indicator of developing intestinal hypoperfusion prior to the clinical manifestation or definitive radiographic evidence and may help in early diagnosis.^23^ The high lactate levels are also indicative of the extent and severity of tissue injury, systemic inflammatory response, and multiorgan dysfunction, which may help identify patients at elevated risk for disease progression, surgical intervention, and mortality.^20^

### Limitations:

Firstly, the number of studies in the review was very relatively small. Due to this we could not conduct a comprehensive subgroup analyses or meta-regressions to assess the source of heterogeneity. Secondly, high heterogeneity was noted in the included studies They included different patient populations, there was difference ins NEC severity, timing of lactate assessment, lactate subtypes, and statistical techniques, all of which may have affected pooled estimates. This limits the applicability of the findings across various clinical settings. Thirdly, the predominance of retrospective study designs is associated with potential biases related to patient selection and residual confounding. Fourth, lactate levels were frequently measured at only a single time point. Only serial assessments can provide more detailed information related to disease progression and prognosis. Fifth, the present analysis combined studies reporting HR and OR which may not be exactly coherent since HR is a time-dependent assessment. The present analysis also combined studies with lactate as a continuous and categorical thresholds. These approaches also capture different aspects of risk and are not directly comparable. This may have contributed to the observed heterogeneity and influenced the pooled estimates. Therefore, the findings of this meta-analysis should be regarded as exploratory rather than confirmatory. Lastly, due to the small number of studies, we could not assess publication bias.

### Strengths of the study:

It is the first study in literature to provide combined evidence on the ability of lactate to discriminate NEC and also synthesis evidence on its prognostic role. The results of this meta-analysis indicate that blood lactate levels may function as a supplementary biomarker while assessing neonates suspected of or diagnosed with NEC. Elevated lactate concentrations could help physicians as well as nursing personnel to identify infants at increased risk of disease progression and adverse outcomes when evaluated with clinical findings and imaging results. Nonetheless, due to heterogeneity among studies and the limited diagnostic information on lactate, it should not be used as the sole diagnostic criterion for NEC. Instead, its primary clinical application should be to stratify risk and monitor disease progression, especially via serial assessments that guide prompt escalation of treatment and surgical decision-making. Given its simplicity, nursing personnel can incorporating lactate measurements into their domain that could improve early detection and prediction of clinical outcomes in NEC.

## CONCLUSIONS

This review suggests that lactate levels are significantly higher in neonates with NEC as compared with controls. However, while elevated lactate tends to be associated with increased mortality risk, substantial heterogeneity and limited evidence preclude its use as a standalone prognostic marker. Further research is needed to strengthen the present conclusions.

### Recommendations:

Further, prospective studies providing data on diagnostic accuracy are needed to assess the sensitivity and specificity of lactate for diagnosis of NEC. Future studies should also standardize data analysis and provide adjusted data to better elucidate the prognostic ability of lactate for NEC.

### Authors’ contributions:

**HS:** Literature search, study design and manuscript writing.

**HS and YL:** Data collection, data analysis and interpretation. Critical Review.

**HS:** Manuscript revision and validation and is responsible for the integrity of the study.

All authors have read and approved the final manuscript.
